# The Critical Role of IL-15–PI3K–mTOR Pathway in Natural Killer Cell Effector Functions

**DOI:** 10.3389/fimmu.2014.00187

**Published:** 2014-04-23

**Authors:** Neethi Nandagopal, Alaa Kassim Ali, Amandeep Kaur Komal, Seung-Hwan Lee

**Affiliations:** ^1^Department of Biochemistry, Microbiology and Immunology, Faculty of Medicine, University of Ottawa, Ottawa, ON, Canada

**Keywords:** natural killer cells, IL-15, JAK–STAT pathway, signal transduction, mTOR pathway

## Abstract

Natural killer (NK) cells were so named for their uniqueness in killing certain tumor and virus-infected cells without prior sensitization. Their functions are modulated *in vivo* by several soluble immune mediators; interleukin-15 (IL-15) being the most potent among them in enabling NK cell homeostasis, maturation, and activation. During microbial infections, NK cells stimulated with IL-15 display enhanced cytokine responses. This priming effect has previously been shown with respect to increased IFN-γ production in NK cells upon IL-12 and IL-15/IL-2 co-stimulation. In this study, we explored if this effect of IL-15 priming can be extended to various other cytokines and observed enhanced NK cell responses to stimulation with IL-4, IL-21, IFN-α, and IL-2 in addition to IL-12. Notably, we also observed elevated IFN-γ production in primed NK cells upon stimulation through the Ly49H activation receptor. Currently, the fundamental processes required for priming and whether these signaling pathways work collaboratively or independently for NK cell functions are poorly understood. To identify the key signaling events for NK cell priming, we examined IL-15 effects on NK cells in which the pathways emanating from IL-15 receptor activation were blocked with specific inhibitors. Our results demonstrate that the PI3K–AKT–mTOR pathway is critical for cytokine responses in IL-15 primed NK cells. Furthermore, this pathway is also implicated in a broad range of IL-15-induced NK cell effector functions such as proliferation and cytotoxicity. Likewise, NK cells from mice treated with rapamycin to block the mTOR pathway displayed defects in proliferation, and IFN-γ and granzyme B productions resulting in elevated viral burdens upon murine cytomegalovirus infection. Taken together, our data demonstrate the requirement of PI3K–mTOR pathway for enhanced NK cell functions by IL-15, thereby coupling the metabolic sensor mTOR to NK cell anti-viral responses.

## Introduction

Natural killer cells are innate immune lymphocytes that were so named for their propensity to kill target cells without the need for antigenic stimulation (1). They are equipped with several inhibitory receptors that bind to certain surface molecules like self-major histocompatibility complex (MHC) class I thereby avoiding destruction of healthy cells. Known as “missing self” hypothesis, NK cells can directly lyse target cells with reductions in or deficiency of MHC class I molecules (2). In addition, signals through activating receptors stimulate NK cells to release pre-existing cytotoxic granules such as perforin and granzymes thereby destroying infected and cancerous cells. Simultaneously, the signals also induce production of inflammatory cytokines such as IFN-γ and TNF-α (3). NK cell effector responses are often determined by the integration of signal transductions pathways from multiple activating and inhibitory receptors (4, 5). Even though NK cells were originally recognized as “ready to act” cells that can immediately cope with virus-infected or transformed cells (6, 7), NK cells derived from laboratory strains of mice housed in specific pathogen-free (SPF) environment show minimal effector functions (8–10). Unlike human NK cells, naïve mouse NK cells are devoid of perforin and granzyme B cytotoxic granules and require additional stimulations to induce rapid translation of their pre-existing mRNAs in order to be fully equipped for action (9).

Several cytokines have been identified to provide such additional stimulations, interleukin-15 (IL-15) being most potent among them in inducing NK cell homeostasis, survival, and maturation. Studies have shown that IL-15^−/−^ mice exhibit selective loss of memory phenotype CD8^+^ T cells, NKT cells, and NK cells (11–13). IL-15R is a heterotrimeric receptor consisting of a unique α chain, a shared β subunit with IL-2, and a common γ subunit with several cytokines (12). A pivotal role of dendritic cell (DC)-expressed IL-15 receptor α chain (IL-15Rα) for trans-presenting IL-15 to NK cells has been demonstrated (14, 15). During inflammation, NK cells are recruited to lymph nodes where they are activated by trans-presentation of IL-15 by IL-15Rα expressed on DCs (8). Engagement of IL-15R on NK cells causes auto-phosphorylation and activation of Janus kinases (JAK1 and JAK3), which induces at least three parallel signaling cascades: Ras–Raf–MEK, PI3K–AKT–mTOR, and signal transduction and activation of transcription (STAT) 5 pathways (12, 16). Data from *Stat5* knock-out and NK cell-specific *Stat5* knock-out mice showed that NK cells are absent in peripheral lymphoid organs, suggesting a critical importance of the IL-15–STAT5 pathway in NK cell development (17–19). In addition, similar to STAT5 knock-out mice, a severe reduction in NK cell numbers has been found in a patient containing the *STAT5b* mutation (20). Studies have shown that IL-15 activates NK cells to become equipped with cytotoxic granules and sensitize them to secondary stimuli. This “priming” has been previously demonstrated with respect to IL-12 and IL-15 co-stimulation, which induces an exaggerated IFN-γ response in NK cells (8, 21, 22). However, it is largely unknown if one of three major signaling pathways is responsible for NK cell priming or it is achieved by a collaborative effort of multiple pathways.

In this study, we set out to investigate the signaling pathway downstream of IL-15 stimulation responsible for sensitizing NK cells to subsequent stimulations. We hypothesized that IL-15-mediated priming of NK cells is not restricted to IL-12 stimulation, but can be extended to other cytokines. Our data indicated that prior exposure to IL-15 dramatically increased NK cell responses to stimulations though Ly49H activation receptor in addition to a myriad of cytokine receptors that employ the JAK–STAT pathway. Furthermore, we show that PI3K–mTOR pathway is crucial for major effector functions in addition to the IL-15-mediated priming process for cytokine responses in NK cells. To translate the importance of PI3K–mTOR pathway for NK cell functions *in vivo*, we treated mice with rapamycin to block mTOR kinase activity downstream of PI3K (23, 24). Interestingly, we observed attenuated anti-viral responses by NK cells upon challenge with murine cytomegalovirus (MCMV) in rapamycin-treated mice. Taken together, our results demonstrate that the PI3K–AKT–mTOR pathway is central to IL-15-induced activation of vital NK cell functions.

## Materials and Methods

### Mice, MCMV infection, and *in vivo* rapamycin treatments

WT C57BL/6 and B6.SJL (C57BL/6 congenic mice with CD45.1 allotype marker) mice from Charles River were housed in SPF environment and used for experiments at 7–12 weeks of age. All procedures were approved by and conducted in accordance with the institution’s animal guidelines of the University of Ottawa. Smith strain MCMV stocks were generated in our laboratory from infected salivary glands of BALB/c mice and viral titers determined by standard plaque assays. WT C57BL/6 mice were infected with 5,000 plaque forming unit (PFU) of MCMV intraperitoneally 4 h after first rapamycin injection. Rapamycin (3 mg/kg/day) or DMSO as vesicle control was administered through intraperitoneal injections once per day until sacrificed.

### Reagents and antibodies

The following monoclonal antibodies were used: α-CD16/32 (clone 2.4G2) from Bioexpress, α-human/mouse Granzyme B (clone GB12) and fixable far red live/dead from Invitrogen. α-Ly49H (clone 3D10), α-TCR-β (clone H57-597), α-NK1.1 (clone PK136), α-CD49b (clone DX5), α-CD8a (clone 53-6.7), and α-IFN-γ (clone XMG1.2) from eBiosciences, α-BrdU (clone B44), α-CD4 (clone RM4-5), and mouse isotype IgG-κ from BD Biosciences. For detection of phosphorylated signals, BD PhosFlow antibodies against pSTAT1 (clone 49), pSTAT3 (clone 4), pSTAT4 (clone 38), pSTAT5 (clone 47), and pSTAT6 (clone 18) were used except α-pS6 ribosomal protein (Ser235/236) (clone D57.2.2E) from Cell Signaling. Cytokines, recombinant murine (rm) IL-2, rmIL-4, rmIL-12, rmIL-15/IL-15Rα complex, and rmIL-21, are from eBiosciences except rmIFN-α from Miltenyi Biotec. To physiologically mimic trans-presentation of IL-15 to NK cells by DCs *ex vivo*, we decided to use rmIL-15/IL-15R complex to enable IL-15 stimulation of NK cells. The rmIL-15/IL-15R complex concentration of 10 ng/ml was determined to induce maximum response in NK cells without inducing equivalent stimulation on CD8 T cells. The following inhibitors were purchased from Calbiochem and used at the indicated concentrations; JAK inhibitor I (0.4 μM), STAT5 inhibitor III pimozide (10 μM), PI3K inhibitor LY294002 (4 μM), mTOR inhibitor rapamycin (1.6 μM *in vitro*; 3 mg/kg *in vivo*), AKT inhibitor VIII AKTi-1/2 (0.8 μM), and MEK inhibitor PD98059 (20 μM).

### Cell isolation and stimulation

Spleens were harvested under sterile conditions, and single cell suspension of splenic leukocytes was generated after red blood cell lysis and filtration through 70 μm nylon mesh. Cells were cultured in RP-10 media (RPMI-1640 from HyClone, 10% FCS, 10 mM HEPES, 1× penicillin/streptomycin, 1% l-Glutamine, 50 μM β-mercaptoethanol) at 37°C and 5% CO_2_. To block signaling molecules, 1 × 10^6^ splenic leukocytes were incubated in 96-well plates (2 × 10^7^/ml in 150 μl) in triplicates with the inhibitors for 1 h prior to stimulation with rmIL-15/IL-15Rα complex (10 ng/ml). For evaluation of STAT activation, cells were harvested 24 h later, washed, and rested for 4 h in RP-10 media (without IL-15) to remove the reversible cell-permeable inhibitors and the residual effects of IL-15 on STAT activation. This was followed by stimulation with rmIL-21 (100 ng/ml), rmIL-12 (10 ng/ml), rmIFN-α (1,000 U/ml), rmIL-4 (100 ng/ml), or rmIL-2 (50 ng/ml) for 30 min before harvesting for flow analyses. The working concentrations of the cytokines were determined to induce maximal responses in NK cells.

### Flow cytometry

For detection of phosphorylated STATs (pSTATs) and S6 ribosomal protein, cells were blocked with 2.4G2 and surface stained for NK and T cells with monoclonal antibodies specific for NK1.1 and TCR-β. Following fixation with BD Cytofix/Cytoperm buffer and permeabilization with pre-chilled 100% methanol, cells were stained for respective intracellular pSTATs or pS6, as previously reported (25). Alternatively, cells were surface stained for NK and T cell proportions followed by staining for intracellular Granzyme B expression immediately after 24 h of IL-15 stimulation. Similarly, cells were recovered after 24 h of IL-15 treatment and surface stained for NK and T cells along with the live/dead viability marker to measure the extent of cell toxicity after inhibitor treatment. NK cell proliferation was measured at 42 h (*ex vivo*) post treatment with 10, 50, or 100 ng/ml of IL-15/IL-15Rα complex or 2.5 days (*in vivo*) post-MCMV infection. BrdU (200 μM) was added to cultures (*ex vivo*) or given as i.p. injections (*in vivo*) 2 h prior to intracellular staining for BrdU incorporation with anti-BrdU antibody (26).

To measure IFN-γ production, IL-15/IL-15Rα complex-treated cells were added to 96-well plates with plate bound α-Ly49H or control IgG antibody (both 10 μg/ml). Alternatively, IL-15/IL-15Rα complex-treated cells were added to 96-well plates with either media alone or rmIL-12 (50 ng/ml). Cells were stimulated for 5 h, last 4 h in the presence of 5 μg/ml brefeldin A, harvested and stained for intracellular IFN-γ expression. For intracellular IFN-γ measurements *in vivo*, splenic leukocytes were harvested post-infection at D1.5 and incubated in media containing brefeldin A in addition to DMSO or rapamycin (1.6 μM) for 4 h, followed by staining for intracellular IFN-γ. In all conditions, freshly isolated untreated naïve total splenic leukocytes were used as controls. All intracellular procedures were carried out using BD Cytofix/Cytoperm protocols. Cells were suspended in staining buffer (1× PBS, 2%FCS, and 0.09% sodium azide), acquired using FACS Cyan ADP and analyzed with Kaluza software v2 (Beckman Coulter).

### Plaque and cytokine quantification assays

For measuring viral burdens in organs, spleens and livers from infected mice were homogenized by Magna lyser (Roche Applied Science) and the lysates were appropriately diluted and overlaid on mouse embryonic fibroblasts cells in duplicates in 10% Dulbecco Modified Eagle’s Medium (DMEM) (DMEM from HyClone, 10% FCS, 10 mM HEPES, 1× penicillin/streptomycin, 1% l-Glutamine). Plaques were counted 3 days later and represented as log PFU/organ. Blood serum and lysates of homogenized spleen and livers from mice were appropriately diluted and analyzed for the production of mouse IFN-γ using Cytometric Bead Array kit (BD Biosciences). Samples were prepared according to manufacturer’s instructions, acquired on FACS Cyan ADP (Beckman Coulter) and analyzed using the BD FCAP Array Software (BD Biosciences).

### NK cell cytotoxicity assay

Natural killer cell cytotoxicity was measured by analyzing the anti-tumor activity of NK cells against YAC-1 target cells. Splenic leukocytes were isolated from infected and control mice treated with either DMSO or rapamycin. Spleen populations were surface stained for NK and T cells to identify NK cell proportions and was adjusted to ensure that equal numbers of NK cells were used in assays at the indicated effector cell (NK cell) to target ratio. Each adjusted sample was prepared in sterile NK media (RPMI-1640 from HyClone, 10% FCS, 10 mM HEPES, 1× penicillin/streptomycin, 1% l-Glutamine, 50 μM β-mercaptoethanol, 1% non-essential amino acids, 1 mM sodium pyruvate) containing either DMSO or 1.6 μM rapamycin. YAC-1 cells were cultured in sterile RP-10 media and labeled with 100 μCi of ^51^Cr for 1 h at 37°C, washed three times with PBS, and made to a concentration of 5 × 10^4^ cells/ml in NK medium. In V-bottom 96-well plates, 100 μl of YAC-1 cells were added in triplicates and incubated for 4 h with 100 μl of effector cells with NK cell: target ratios ranging from 0.3:1 to 10:1. Supernatants were then used to quantify the amount of ^51^Cr released due to specific lysis of YAC-1 targets by NK cells, and counted using 2470 WIZARD^2^ Automatic Gamma Counter (Perkin Elmer). NK cytotoxicity was calculated according to the formula: % specific ^51^Cr release = [(experimental release − minimum release)/(maximum release − minimum release)] × 100.

### RNA extraction and quantitative PCR

Total RNA was extracted from splenic leukocytes using TRIZOL reagent (GIBCO BRL) according to manufacturer’s instructions. cDNA was reverse transcribed from 500 ng of total RNA in a 20-μl reaction using first strand cDNA synthesis kit (Thermo Scientific). For quantification of β-actin and IL-15 genes by real-time PCR, one-tenth volume of cDNA was added to a 15-μl reaction of FastStart Universal Probe Master Kit (Roche) and amplified using ViiA 7 Dx real-time PCR Instrument (Applied Biosystems). Expression of IL-15 in all the samples was normalized to β-actin levels. The primer sequences for β-actin are forward: 5′CCAACCGTGAAAAGATGAC3′ and reverse: 5′GTACGACCAGAGGCATACAG3′, for IL-15 are forward: 5′ACATCCATCTCGTGCTACTTGT3′ and reverse: 5′GCCTCTGTTTTAGGGAGACCT3′ (27).

### Statistics

Significance of results was determined by two-tailed unpaired student *t* tests (*p ≤ 0.05; **p ≤ 0.01; ***p ≤ 0.001) and graphed using Graph Pad Prism 5 software.

## Results

### IL-15 primes NK cells through a myriad of cytokine receptors and Ly49H activation receptor

In addition to inhibitory and activating receptors, NK cells express receptors for various cytokines. Because the priming effect has only been shown with respect to enhanced responsiveness to IL-12 when co-stimulated with IL-2 or IL-15 (8, 21, 22, 28–30), we hypothesized that IL-15 “priming” of NK cell responses can be extended to a broader range of cytokines that transmit their signal by employing the JAK–STAT pathway. Signal transduction following cytokine stimulation involves multiple pathways, making it difficult to determine whether functional consequences result from direct or indirect effects. Therefore, to measure direct responses to cytokine stimulation, we decided to evaluate the level of pSTATs, an activation event occurring proximal to cytokine receptors.

**Figure 1:**
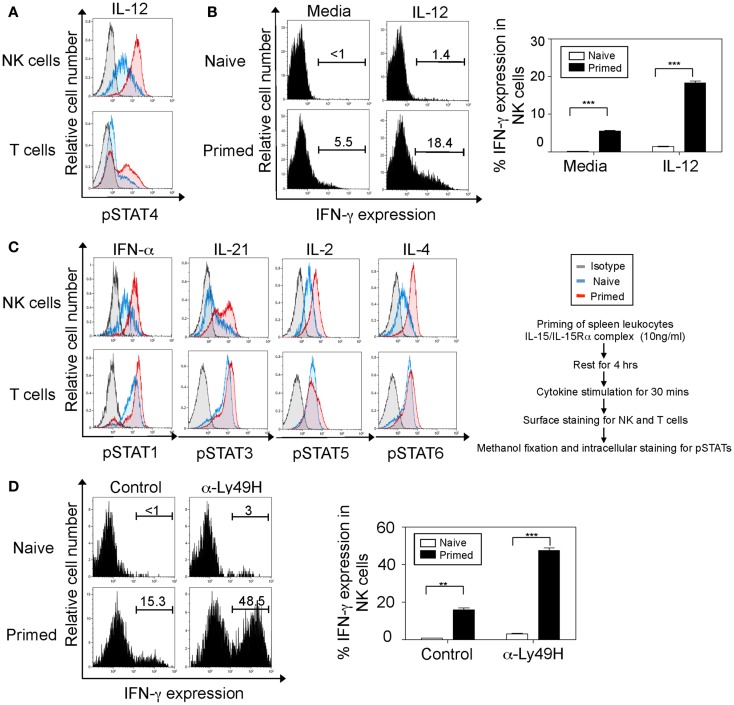
**Interleukin-15 primes NK cells through a broad range of cytokine receptors and the Ly49H activation receptor**. NK cells were defined as NK1.1^+^TCRβ^−^populations and T cells as NK1.1-TCRβ^+^ populations. **(A)** Naïve and IL-15 primed splenic leukocytes (for 24 h) were stimulated with IL-12 for 30 min followed by intracellular staining for phosphorylated STAT4 protein. Histograms depict the expression of phosphorylated STAT4 protein in NK and T cells. Naïve cells are depicted in blue, IL-15-treated cells in red, and isotype antibody (negative controls for intracellular staining) in gray. **(B)** Naïve and IL-15 primed splenic leukocytes were stimulated with media or IL-12 for 5 h; brefeldin A was added during the last 4 h. Cells were then stained for intracellular IFN-γ. Histograms depict IFN-γ expression upon IL-12 stimulation in NK cells and summarized in graphs where each bar indicates an average of duplicate samples. **(C)** Naïve and IL-15 primed splenic leukocytes (for 24 h) were stimulated with the indicated cytokines. After 30 min, cells were stained for intracellular expression of phosphorylated STAT proteins. Histograms depict the phosphorylation of respective STAT proteins in NK and T cells. **(D)** Naïve and IL-15 primed splenic leukocytes were stimulated on α-Ly49H or control antibody coated plates for 5 h; brefeldin A was added during the last 4 h. Cells were then stained for intracellular IFN-γ. Histograms depict IFN-γ expression upon Ly49H or control IgG1 stimulation in NK1.1^+^TCRβ^−^cells and summarized in graphs where each bar indicates an average of duplicate samples. Figures are representative of three independent experiments. Numbers on histograms indicate proportions of IFN-γ-expressing cells. Significance of results was determined between naïve and primed cells. **p ≤ 0.01; ***p ≤ 0.001.

IFN-γ production in NK cells is regulated by IL-12 in a STAT4-dependent manner (31, 32). Therefore, we first set out to analyze the sequential responses to IL-12 stimulation (rather than co-stimulation) in NK cells that have been previously exposed to IL-15. Indeed, our data indicate that NK cells pre-stimulated with IL-15 display enhanced phosphorylation of STAT4 and consequently increased IFN-γ production upon IL-12 stimulation compared to naïve cells (Figures 1A,B). To test whether IL-15 can prime NK cells for an extensive list of cytokines in addition to IL-12, we have stimulated naïve and IL-15/IL-15Rα complex-primed splenic leukocytes with five representative cytokines (i.e., Type I IFN, IL-21, IL-12, IL-2, and IL-4), which transmit their signals through STAT1, STAT3, STAT4, STAT5, and STAT6 molecules respectively (33). Notably, our results demonstrated that in addition to previously described IL-12 effect, IL-15 can prime NK cells to stimulations with all cytokines tested based on enhanced pSTAT expression (Figure 1C). It is worth noting that this priming effect was predominantly observed in NK cells compared to T cells.

We next analyzed whether this increased responsiveness in primed NK cells can be extended to stimulation through activation receptors. It is known that stimulation through both IL-12–STAT4 and Ly49H–Syk/ZAP70 receptors induces IFN-γ production in NK cells (3–5). We therefore analyzed the IFN-γ expression in primed NK cells after stimulation through Ly49H activation receptor. Our results indicate that primed NK cells display significantly elevated IFN-γ expression in NK cells when stimulated though their Ly49H receptor compared to naïve cells (Figure 1D). Even though stimulations induced basal IFN-γ production in naïve NK cells, IL-15-primed NK cells produced dramatically enhanced level of IFN-γ. Taken together, IL-15-priming sensitizes NK cells to stimulations through cytokine receptors and Ly49H activation receptor.

### Requirement of PI3K–mTOR pathway in primed NK cell responses to cytokine stimulation

IL-15 binding to its receptor initiates signaling through activation of JAK 1/3, which induce at least three parallel cascades: Ras–Raf–MEK, PI3K–AKT–mTOR, and STAT5 (12, 16). In order to identify which signaling pathway downstream of IL-15–JAK activation is predominately responsible for NK cell priming, we prepared NK cells in which each pathway is abrogated by pretreatment with specific cell-permeable inhibitors. We used PD98059 for blocking ERK 1/2, LY294002 for blocking PI3K, and pimozide (STAT5 inhibitor III) for blocking STAT5. In addition, AKT-1/2 inhibitor (CAS 612847-09-3) and rapamycin (mTOR inhibitor) were used to investigate the signaling events downstream of PI3K pathway.

**Figure 2:**
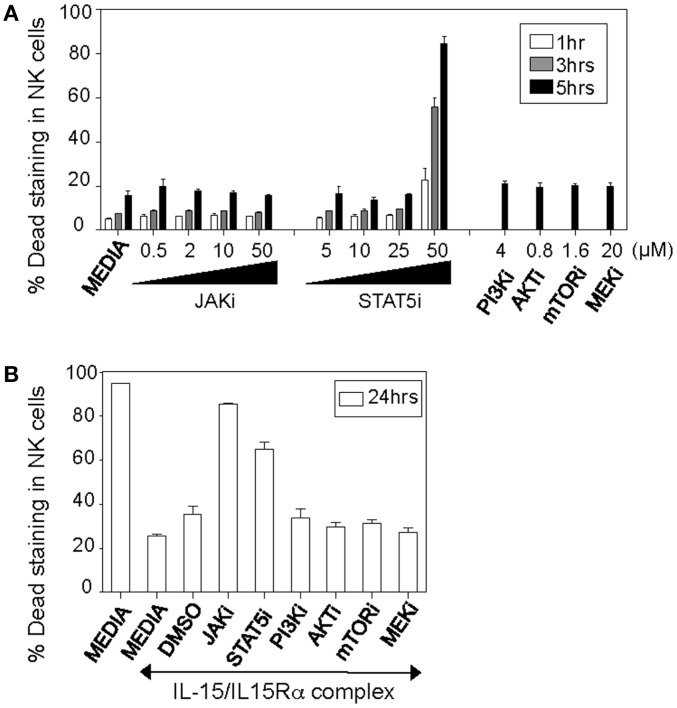
**Effect of various inhibitors on NK cell viability**. **(A)** Naïve splenic leukocytes were incubated with indicated concentrations of JAK and STAT5 inhibitors for 1, 3, and 5 h, in addition to media alone or working concentrations of the other inhibitors for 5 h. Toxicity of the inhibitors was analyzed by staining for proportions of live/dead cells among various populations. Graph depicts percentages of NK cells that stain positive for dead staining. **(B)** Similarly, splenic leukocytes were incubated with working concentrations of the inhibitors (see Materials and Methods) or DMSO controls for 1 h, following which cells were cultured for 24 h in media with or without IL-15/IL-15Rα. Graph depicts percentages of IL-15-treated NK cells that stain positive for dead staining. NK cells were defined as NK1.1^+^TCRβ^−^populations. Figures are representative of at least three independent experiments. Each bar indicates an average of duplicate samples.

To optimize the conditions for each inhibitor at which IL-15 priming can be achieved without compromising cell viability, we analyzed toxicity in NK cells within a few hours of inhibitor treatment. Notably, we observed that NK cell deaths in samples with working concentrations of inhibitors were comparable to controls (without any inhibitor treatment), suggesting no direct toxicity contributed by inhibitors (Figure 2A). In addition, NK cells were also measured for cell death after 24 h of IL-15 stimulation. Cells were treated with inhibitors for 1 h followed by IL-15/IL-15Rα complex treatment. Since IL-15 is essential for NK cell survival, cells deprived of IL-15 exhibit drastic cell deaths (Figure 2B). JAK1/3–STAT5 has been known for mediating pro-survival effects of IL-15 in the cell (12, 34, 35). As expected, blocking JAK and STAT5 pathway resulted in dramatic cell deaths similar to NK cells deprived of IL-15. Notably, all other inhibitors tested maintained NK cell viability compared to IL-15-treated controls (cells without any inhibitor treatment), suggesting that blocking of PI3K and MEK pathways at the indicated inhibitor concentrations do not affect NK cell survival.

**Figure 3:**
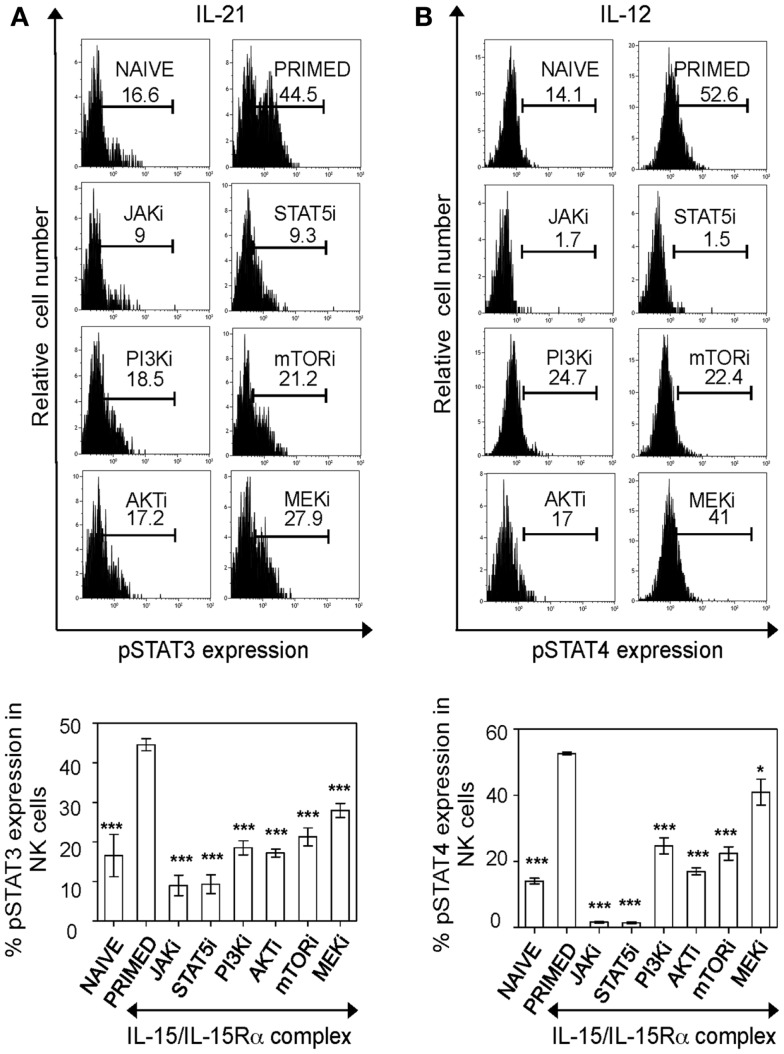
**PI3K–AKT–mTOR pathway is critical for responsiveness of primed NK cells to cytokine stimulation**. Splenic leukocytes were incubated with working concentrations of the inhibitors for 1 h; followed by culturing of cells for 24 h with IL-15/IL-15Rα. Naïve and inhibitor treated-primed splenic leukocytes were washed and rested for 4 h followed by stimulation with the indicated cytokines. After 30 min of cytokine stimulation, cells were stained for intracellular phosphorylated STAT proteins. Histograms depict expression of phosphorylated STAT3 and STAT4 proteins in primed NK cells upon stimulation with IL-21 **(A)** and IL-12 **(B)** respectively and the effect of various inhibitors on the same. Results are summarized in graphs where each bar indicates an average of triplicate samples. Figures are representative of at least three independent experiments. Numbers on histograms indicate percentages of pSTAT expression in cells. NK cells were defined as NK1.1^+^TCRβ^−^populations. Significance of results was determined with IL-15 primed cells without any inhibitor treatment. *p ≤ 0.05;***p ≤ 0.001.

Having established the optimal concentration of inhibitors, we investigated the major pathway responsible for cytokine responsiveness of IL-15 primed NK cells. IL-15-induced pathways were blocked in naïve splenic leukocytes by adding inhibitors 1 h before IL-15/IL-15α complex stimulation for a total of 24 h. Treated cells were washed and rested for 4 h to remove residual effects of IL-15 and inhibitors. They were then evaluated for phosphorylated STAT3 and STAT4 upon the stimulation with IL-12 and IL-21, respectively. Blocking of either JAK or STAT5 abrogated pSTAT expression, however it can be attributed to negative effect of those inhibitors on NK cell survival. Notably, treatment with PI3K inhibitor abrogated the priming effect, with pSTAT levels similar to that of naïve cells. Such inhibition was also observed with blocking mTOR and AKT, downstream signaling components of PI3K pathway, suggesting that PI3K–AKT–mTOR pathway is critical for optimal responses of “primed” NK cells to cytokine stimulations (Figures 3A,B). Treatment of naïve cells with the inhibitors did not show any reduction in their cytokine responsiveness compared to controls (data not shown), indicating that the pathway is specifically implicated in IL-15 primed NK cells. In addition, blocking MEK reduced pSTAT levels significantly with IL-21 and modestly with IL-12 stimulations. Therefore, IL-15 treatment enhances NK cell responsiveness to IL-12 and IL-21 stimulations and the effects of priming is abrogated by blocking PI3K–AKT–mTOR pathway during IL-15 treatment.

### PI3K–mTOR pathway is implicated in IL-15-induced effector functions of NK cells

**Figure 4:**
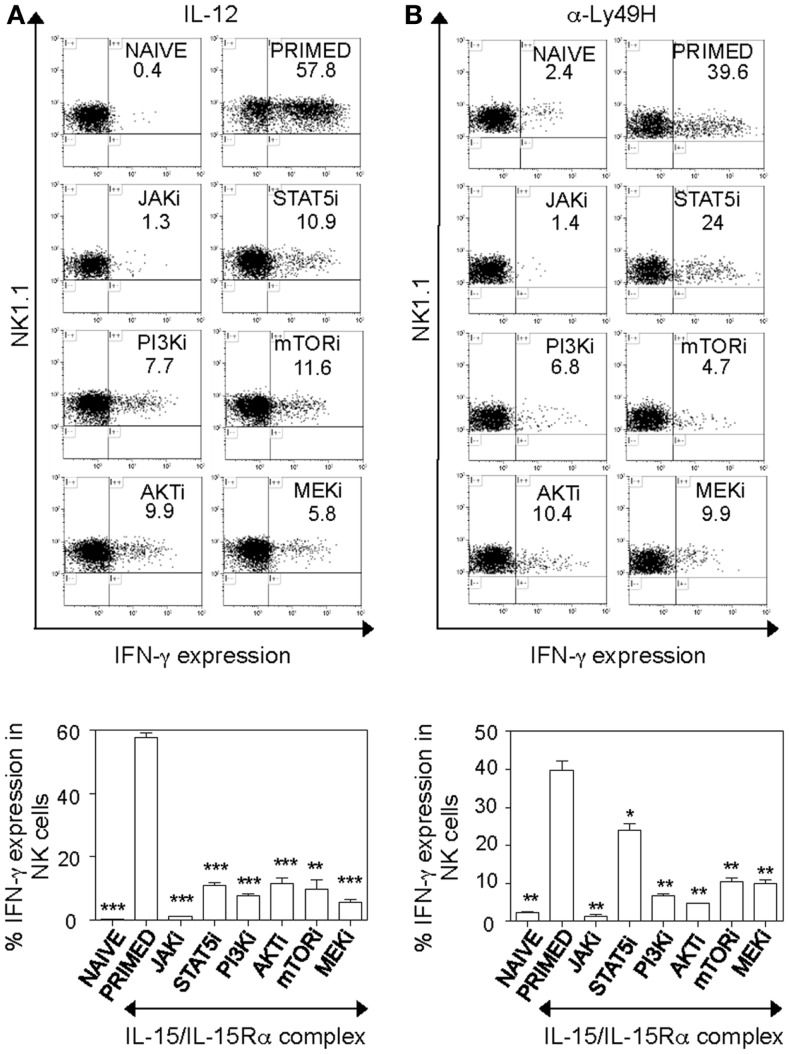
**PI3K–AKT–mTOR pathway is required for optimal IFN-γ production in primed NK cells**. Splenic leukocytes were incubated with working concentrations of the inhibitors for 1 h; followed by culturing of cells for 24 h with IL-15/IL-15Rα. Naïve and inhibitor treated-primed splenic leukocytes were stimulated with IL-12 or in α-Ly49H coated plates for 5 h; Brefeldin A was added in the last 4 h. Cells were then stained for intracellular expression of IFN-γ. Histograms depict IFN-γ expression in NK cells upon stimulation with IL-12 **(A)** and plate-bound α-Ly49H **(B)** respectively and the effect of various inhibitors on IFN-γ expression. Results are summarized in graphs where each bar indicates an average of duplicate samples. Figures are representative of two independent experiments. Numbers on histograms indicate proportions of IFN-γ-expressing NK cells. NK cells were defined as NK1.1^+^TCRβ^−^populations. Significance of results on each bar was determined with primed cells without any inhibitor treatment. *p ≤ 0.05; **p ≤ 0.01;***p ≤ 0.001.

During inflammation, NK cells are exposed to IL-15 at early stages in lymph nodes and become activated and recruited to sites of inflammation in the periphery (8) where they can produce pro-inflammatory cytokines like IFN-γ and kill target cells via perforin/granzyme-mediated cytotoxicity. Therefore, we decided to evaluate whether the PI3K–AKT–mTOR pathway is also required for NK cell effector functions induced by IL-15. Consistent with previous data, IL-15 primed NK cells produced significantly more IFN-γ than naïve cells upon IL-12 and α-Ly49H stimulation. Interestingly, IFN-γ production in both stimulations was severely reduced by PI3K–AKT–mTOR inhibition (Figures 4A,B). Blocking of JAK abrogated IFN-γ production, however as observed previously (Figure 2B), this could be due to the negative effect of JAK inhibitor on NK cell survival after 24 h of incubation. Blocking STAT5 reduced IFN-γ production upon IL-12 stimulation of NK cells, but STAT5 inhibitor only moderately affected IFN-γ production in NK cells with α-Ly49H stimulation. This suggests that unlike in IL-12 stimulation, blocking STAT5 may not be detrimental for primed NK cell responses to α-Ly49H stimulation. In addition, MEK inhibition in NK cells reduced IL-15-induced elevation in IFN-γ production with both cytokine and activation receptor stimulation.

**Figure 5:**
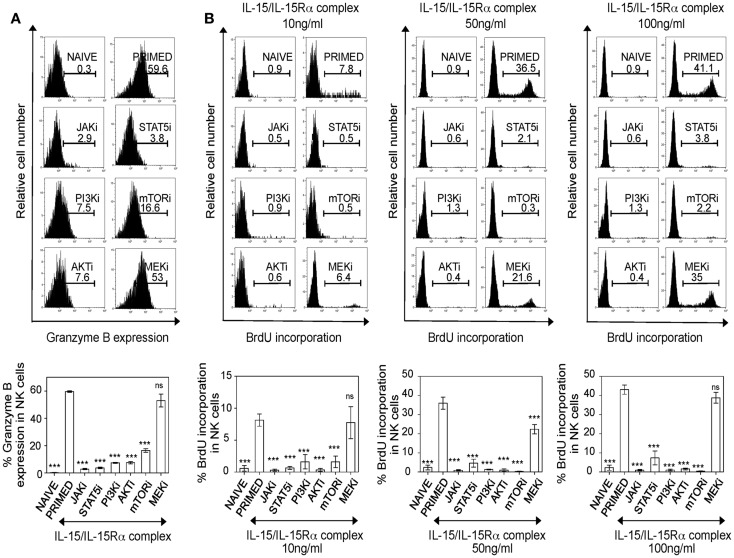
**PI3K–AKT–mTOR pathway is required for granzyme B production and proliferation of IL-15-treated NK cells**. **(A)** Splenic leukocytes were incubated with working concentrations of the inhibitors for 1 h; followed by culturing of cells for 24 h with IL-15/IL-15Rα. Naïve and primed cells were stained for intracellular granzyme B. Histograms depict intracellular granzyme B expression in NK cells and the effect of inhibitors on the expression. Results are summarized in graphs where each bar indicates an average of triplicate samples. **(B)** Splenic leukocytes were incubated with working concentrations of the inhibitors for 1 h; followed by culturing of cells for 24 h with IL-15/IL-15Rα at the indicated concentrations. BrdU was added *in vitro* (one-tenth volume of a 96-well) to the cells, 2 h prior to intracellular staining for BrdU. Histograms depict BrdU incorporation in naïve and primed NK cells and the effect of inhibitors on NK cell proliferation at different concentrations of IL-15/IL-15Rα. Results are summarized in graphs where each bar indicates an average of six samples pooled from two independent experiments. Figures are representative of at least three independent experiments. Numbers on histograms indicate proportions of cells expressing granzyme B or percentages of BrdU incorporation in NK cells. NK cells were defined as NK1.1^+^TCRβ^−^populations. Significance of results on each bar was determined with primed cells without any inhibitor treatment. ***p ≤ 0.001.

Unlike human NK cells, NK cells from mouse housed in SPF vivarium are devoid of perforin/granzyme B expression, but can translate the cytotoxic granules from pre-existing mRNA pools upon the activation by IL-15 (9). Consistently, IL-15-treated cells, but not naïve NK cells, expressed significantly more granzyme B (Figure 5A). Blocking PI3K–AKT–mTOR pathway drastically reduced granzyme B expression in IL-15 activated NK cells. Interestingly, MEK did not affect the ability of primed NK cells to express granzyme B.

Since we identified that the PI3K–mTOR pathway is implicated in NK cell effector functions, we next investigated the effect of this pathway in proliferation of NK cells. Splenic leukocytes were stimulated with three different concentrations (10, 50, and 100 ng/ml) of IL-15/IL-15Rα complex and pulsed with BrdU 2 h prior to analysis for BrdU incorporation at 42 h. Consistent with their negative effect on survival, blocking either JAK or STAT5 completely abrogated NK cell proliferation (Figure 5B). Notably, NK cell proliferation was severely affected in PI3K–AKT–mTOR inhibition and this was reproducible in NK cells treated with higher doses of IL-15/IL-15Rα complex. When the effect of MEK inhibition was analyzed at different IL-15 doses, blocking MEK did not abrogate NK cell proliferation with 10 and 100 ng/ml of IL-15/IL-15Rα complex. However, there was decrease in proliferation in NK cells primed with 50 ng/ml of IL-15/IL-15Rα complex, but this was not as dramatic as the effects seen with inhibition of the PI3K–AKT–mTOR pathway. Taken together, our data suggest that PI3K–AKT–mTOR pathway is central for IL-15-mediated NK cell effector responses including granzyme B expression, proliferation, and IFN-γ production. MEK is important for cytokine responses and IFN-γ production but is not critical for granzyme B expression and proliferation; suggesting differential regulation of MEK upon IL-15 stimulation for effector functions of NK cells.

### Requirement of mTOR in MCMV-induced NK cell effector functions

**Figure 6:**
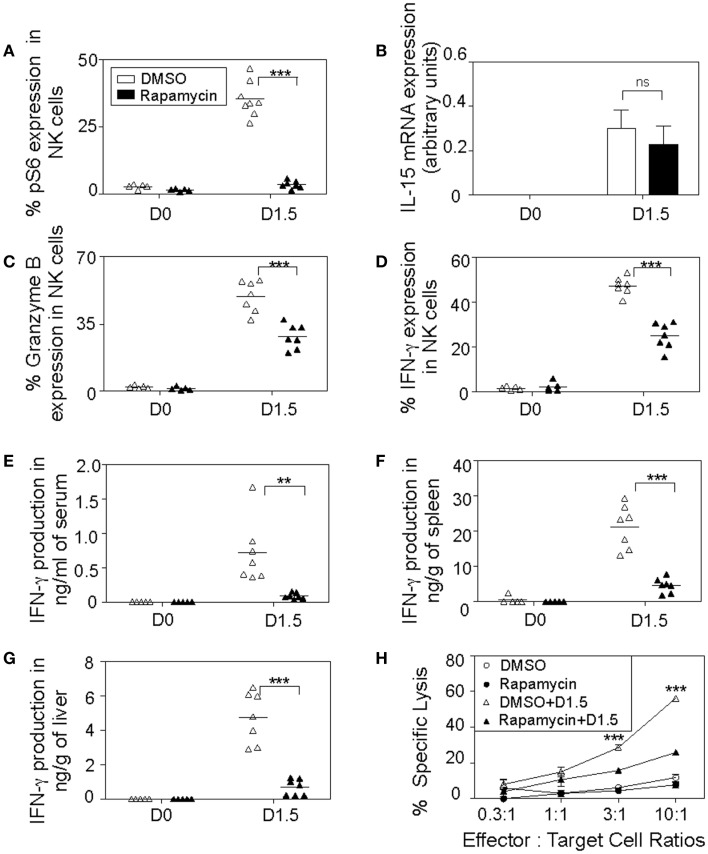
***In vivo* inhibition of mTOR affects NK cell cytotoxic responses**. Rapamycin- or DMSO-treated mice were either uninfected or given 5,000 PFU MCMV i.p. and sacrificed on day 1.5. Splenic leukocytes were isolated and used for the following analyses: **(A)** Graph depicts intracellular expression of phosphorylated S6 ribosomal protein in splenic NK cells. **(B)** Total RNA was extracted and quantified for IL-15 production by real-time PCR. Graph depicts IL-15 mRNA expression in controls and infected samples normalized to β-actin levels. Graphs depict percentages of intracellular granzyme B **(C)** and IFN-γ **(D)** expressions in NK cells. Infected and control organs were isolated, homogenized, and the lysates used for analysis of cytokine production along with blood serum by cytometric bead assays. Graphs depict the amount of IFN-γ levels in serum **(E)**, spleens **(F)**, and livers **(G)**. Data are representative of *n* = 5–7 mice pooled from two independent experiments that were matched for sex and age of mice. **(H)** To measure NK cytotoxic activity, infected and control splenic leukocytes were incubated *ex vivo* with their target YAC-1 tumor cells at the indicated effector (NK cells): target cell ratios for 4 h in media containing DMSO or rapamycin. Graph shows percentages of YAC-1 cells lysed by splenic NK cells where each point indicates an average of triplicate samples. NK cells were defined as NK1.1^+^TCRβ^−^populations. Figures are representative of at least three independent experiments. Significance of results was determined between DMSO and rapamycin-treated samples. **p ≤ 0.01; ***p ≤ 0.001.

To further extend our findings to NK cell functions *in vivo*, we decided to evaluate the effects of blocking mTOR pathway by administering rapamycin during MCMV infection, where critical role of NK cell for early control of virus replication is well known (3, 5). WT B6 mice were given mTOR inhibitor rapamycin or DMSO by intraperitoneal injections daily and either infected with 5,000 PFU MCMV or left uninfected as controls. To confirm inhibition of the mTOR pathway by rapamycin treatment *in vivo*, we evaluated levels of phosphorylated S6 ribosomal protein which is a target of S6 kinase downstream of mTOR pathway (24). Significantly low phosphorylation levels of S6 ribosomal protein were found in rapamycin-treated mice compared to controls, indicating efficient blocking of mTOR pathway by rapamycin (Figure 6A). Next, we evaluated the effects of rapamycin treatment on IL-15 production. IL-15 not only plays a key role in survival, and proliferation, but also functions as a central mediator of inflammation by amplifying the inflammatory response in various immune cells (12). To confirm if rapamycin treatment does not hamper IL-15 production thereby enabling NK cell priming *in vivo*, we quantified IL-15 mRNA levels in splenic leukocytes on day 1.5 by real-time PCR. IL-15 was induced upon MCMV infection and no significant differences in expression levels between rapamycin-treated and control samples were observed (Figure 6B).

We then set out to evaluate effector functions of NK cells during MCMV infections such as intracellular IFN-γ and granzyme B production in rapamycin-treated mice on day 1.5. Both granzyme B (Figure 6C) and IFN-γ (Figure 6D) expression levels were induced after MCMV infection in NK cells. However, their productions were diminished in rapamycin-treated mice compared to controls. At this time point, MCMV infection induced a well-defined innate cytokine peak response of IFN-γ in serum, which was dramatically reduced (>75%), indicating that cytokine response of NK cells is compromised in the rapamycin-treated mice (Figure 6E). We observed similar patterns in the organs, rapamycin-treated mice displayed reduced IFN-γ production in the infected spleens (Figure 6F) and livers (Figure 6G). Additionally, we analyzed NK cell cytotoxic responses *ex vivo* by measuring the ability of NK cells from rapamycin and DMSO-treated control and infected mice to kill the NK cell-sensitive YAC-1 cells. NK cells from infected mice were able to efficiently lyse target cells while naïve cells displayed poor killing activity. Notably, splenic NK cells from rapamycin-treated MCMV-infected mice on day 1.5 had attenuated lytic activities toward target tumor cells (Figure 6H).

**Figure 7:**
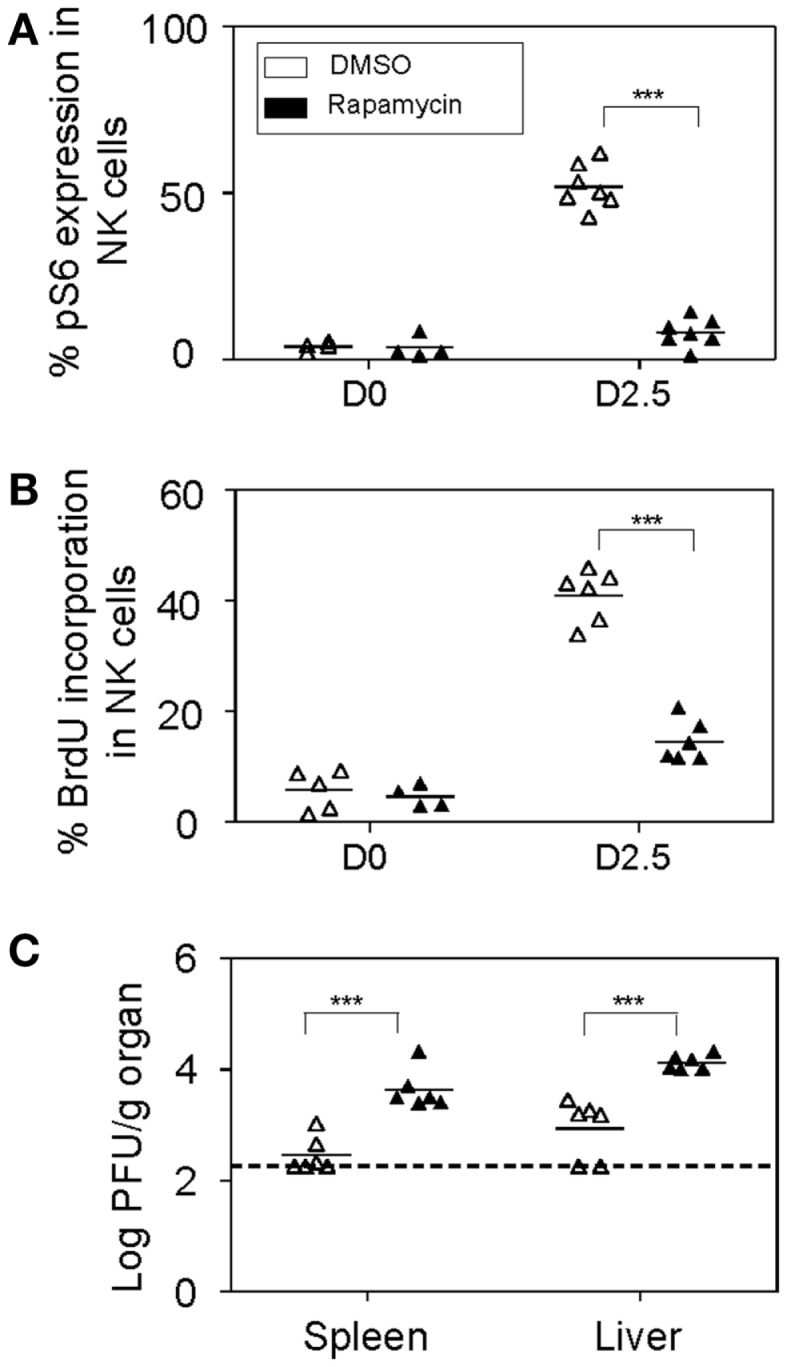
***In vivo* inhibition of mTOR affects NK cell proliferative and anti-viral responses**. Rapamycin or DMSO-treated mice were either uninfected or given 5,000 PFU MCMV i.p. and sacrificed on day 2.5 post-infection for the following analyses: **(A)** Splenic leukocytes were isolated from infected and control organs and analyzed for the effects of rapamycin treatment on mTOR pathway. Graph depicts intracellular expression of phosphorylated S6 ribosomal protein in NK cells. **(B)** BrdU was injected intraperitoneally (2 h prior to sacrifice) following which splenic leukocytes were isolated from infected and control organs and analyzed for NK cell proliferation. Graph depicts proportion of BrdU incorporation in NK cells. **(C)** Infected and control organs were isolated, homogenized, and the lysates used for viral quantification by plaque assays. Graph indicates viral titers in the infected spleens and livers of DMSO or rapamycin-treated mice. Data are representative of *n* = 4–7 mice pooled from two independent experiments that were matched for sex and age of mice. NK cells were defined as NK1.1^+^TCRβ^−^populations. For a given organ showing undetectable plaque, the titer of virus for that organ was arbitrarily set to the limit of detection for statistical calculation and graph representation. Figures are representative of at least three independent experiments. Significance of results was determined between DMSO and rapamycin-treated samples. *p ≤ 0.05; **p ≤ 0.01;***p ≤ 0.001.

As previously mentioned, NK cell anti-viral defenses are critical at early stages for the control of MCMV replication. Therefore, we decided to investigate the consequences of blocking mTOR pathway in NK cells by treating mice with rapamycin and evaluated NK cell responses to MCMV infections on day 2.5. Significant reductions in the phosphorylation of S6 ribosomal protein were observed in MCMV-infected rapamycin-treated mice compared to controls, indicating efficient blocking of the mTOR pathway (Figure 7A). The proportions of NK cells were comparable to controls and unaffected by rapamycin treatment (data not shown). However consistent with our *ex vivo* data, proliferation of NK cells as measured by BrdU incorporation was severely affected in rapamycin-treated mice during infections (Figure 7B). Consequently, when virus loads in organs of mice treated with rapamycin was measured at this time point, significantly higher viral burdens were found in spleens and livers compared to those that received DMSO (Figure 7C). Taken together, blocking mTOR pathway *in vivo* affects cytokine production, and proliferative and cytotoxic responses of NK cells during infections, thereby resulting in elevated viral burdens.

## Discussion

In this report, we have demonstrated that prior exposure to IL-15 primes NK cell responses to further stimulations through a myriad of cytokine receptors and the Ly49H activation receptor. By using specific cell-permeable reversible inhibitors to enable efficient blocking of individual signaling pathways emanating from IL-15 receptor, we further demonstrated that PI3K–mTOR pathway is critical for IL-15-induced major NK cell functions like granzyme B expression, IFN-γ production and proliferation. In addition, our data showed that *in vivo* blocking of mTOR by rapamycin affects NK cell cytotoxic and proliferative responses during MCMV infection, resulting in elevated viral burdens. Taken together, our results show that the PI3K–mTOR pathway is indispensable for efficient NK cell activity and provides an example of how mTOR activity is linked to immune functions of NK cells.

The serine/threonine kinase mTOR is a well-studied regulator of cell growth and metabolism. It acts downstream of the PI3K–AKT pathway and is activated by growth factors, nutrients, and various other signals (23, 36). Activation of mTOR complex 1 (mTORC1) leads to the phosphorylation of several downstream targets such as 70-S6 kinase (S6K); and this kinase activity of mTORC1 is blocked by rapamycin treatment (24). Recently, accumulated evidence demonstrates that mTOR pathway can regulate functions of immune cells by modulating metabolism of the individual immune cells. For example, rapamycin inhibits T cell proliferation and therefore is used as immunosuppressive agent for the prevention of allograft rejection (37). However, treating rapamycin at low doses improves naïve CD8 T cell differentiation to memory CD8 T cells *in vivo*, suggesting immunomodulatory roles in memory response (24). In addition, mTOR-deficient CD4 T cells fail to differentiate into Th1, Th2, and Th17 cells and this defect was largely due to impaired phosphorylation of respective STAT molecules required for each lineage differentiation. This is similar to our *ex vivo* experiments where diminished levels of pSTATs in primed NK cells was observed with rapamycin treatment (38). Here, we show the dramatic effects of rapamycin treatment in NK cells during virus infection. To our knowledge, this is the first study to link the IL-15-induced mTOR pathway to NK cell functions *in vivo* during acute virus infections.

The effect of rapamycin on NK cell functions during MCMV infection was dramatic, showing severe defects in IFN-γ and granzyme B productions in addition to proliferation. It is well known that granzymes and IFN-γ are required for efficient NK cell anti-viral activity (39–42). Consequently, mTOR inhibition affected NK cell cytotoxic responses toward target tumor YAC-1 cells and resulted in elevated viral loads in the infected organs. Presumably, the drastic effect of rapamycin on NK cells is due to the fact that they are the most metabolically active cell population during acute MCMV infection. During infections, NK cells undergo rapid blastogenesis and this expansion is dependent on IL-15 (32, 43). Our data suggest that mTOR pathway might be required for the efficient metabolic transformation of NK cells and the associated immune functions.

PI3K–mTOR pathway is implicated in IFN-γ production by primed NK cells when stimulated through both IL-12 and Ly49H receptors. We analyzed responses of primed NK cells to Ly49H activation receptor, as Ly49H recognition of the viral ligand m157 during MCMV infection triggers NK cell proliferation and enhances anti-viral responses (44–46). However, Ly49H expression in B6 mice strains is restricted to roughly 50% of NK cells; therefore further investigation of NK cell priming to other NK cell activation receptors is warranted. Moreover, several receptors on NK and T cells using different adaptor molecules such as CD3, Syk/ZAP70, DAP10, DAP12, etc. could be employed upstream and possibly converge to a common PI3K–mTOR pathway for NK cell effector functions (47–49).

Previous studies have shown that MEK inhibition affected NK cytotoxic activity by interfering with the mobilization of cytotoxic granules toward target cells in human NK cell lines, while proliferation of NK cells was unaffected (50). This was further shown to be regulated upstream by Syk/ZAP70 molecules associated with the NK activation receptors, that in turn leads to the phosphorylation of PI3K and to the activation of downstream Rac1–PAK1–MEK–ERK molecules (51, 52). The importance of the MEK pathway in NK cell cytokine responses was also reported by another group which demonstrated that the production of pro-inflammatory cytokines like IFN-γ, IL-6, and TNF-α were reduced by MEK blockade in human peripheral blood NK cells. Similarly, MEK was regulated upstream by the activation of Syk–PI3K pathway upon CD160 receptor ligation on NK cells (53). Both these studies are consistent with our data that implicate MEK in responsiveness to IL-12 and IL-21 stimulations and IFN-γ production upon IL-12 and α-Ly49H stimulations, but is not critical for proliferation. Our data show that the intracellular production of granzyme B was unaffected with MEK inhibition, but this does not necessarily translate to efficient NK cytotoxic responses. NK cell activity is regulated at multiple steps and therefore to further understand the effect of MEK inhibition on NK cell lytic activity, the mobilization of cytotoxic granules toward the target cells needs to be analyzed in our model. On the other hand, both *in vivo* and *ex vivo* results clearly show that PI3K–AKT–mTOR is required for granzyme B and IFN-γ productions in NK cells.

We have demonstrated that the signaling axis of IL-15–PI3K–mTOR in NK cells is important for their cellular proliferation, responsiveness to cytokine stimulations, and cytotoxic functions. IL-2/IL-15 share receptor subunits of IL-2/IL-15R-β and -γ chains and both are being widely used for *ex vivo* expansion of NK cells in immunotherapy. Their promising therapeutic capacity for a variety of human malignancies has stimulated an interest in using NK cells for anti-cancer treatments (54, 55). Therefore, understanding the molecular mechanisms by which IL-15 primes and activates NK cells will allow manipulation of IL-15 signaling for improving NK cell-based therapeutic strategies against cancers and infectious diseases.

## Author Contributions

Neethi Nandagopal performed majority of the experiments, analyzed the data, and wrote the manuscript. Alaa Kassim Ali performed the NK killing assay and generated Figure 6H while Amandeep Kaur Komal was involved in designing and performing cytokine detection assays. Seung-Hwan Lee designed the study, supervised the experiments, and wrote the manuscript.

## Conflict of Interest Statement

The Guest Associate Editor Andrew P. Makrigiannis declares that, despite being affiliated to the same institution as authors Neethi Nandagopal, Alaa Kassim Ali, Amandeep Kaur Komal and Seung-Hwan Lee, the review process was handled objectively and no conflict of interest exists. The authors declare that the research was conducted in the absence of any commercial or financial relationships that could be construed as a potential conflict of interest.
